# Esthetic Rehabilitation of a Patient With Amelogenesis Imperfecta Using the Composite Injection Moulding Technique: A Case Report

**DOI:** 10.7759/cureus.87507

**Published:** 2025-07-08

**Authors:** Nadia El Mesbahi, Imane Hachami, El Mehdi Jouhadi, Houda Moussaoui

**Affiliations:** 1 Fixed Prosthodontics Department, Faculty of Dentistry Casablanca, Hassan II University, Casablanca, MAR; 2 Fixed Prosthodontics Department, Centre Hospitalier Universitaire (CHU) Ibn Rochd - Centre de Consultations et de Traitements Dentaires (CCTD), Casablanca, MAR

**Keywords:** amelogenesis imperfecta, composite injection moulding technique, esthetic, mock-up, rehabilitation

## Abstract

Treatment of amelogenesis imperfecta (AI) is considered a challenging treatment. Optimal treatment consists of early diagnosis and a multidisciplinary approach to prevent early destruction by caries and to improve esthetics and oral health-related quality of life.

This study aimed to report on the treatment of a 13-year-old patient with amelogenesis imperfecta (AI) using the composite injection moulding technique to restore the anterior sector. This therapeutic option was chosen based on the patient's age, skeletal discrepancy, and financial considerations.

## Introduction

Amelogenesis imperfecta (AI) describes a group of hereditary characteristics that change the structure and appearance of dental enamel. It is characterized by hypomineralization and/or hypoplasia with discoloration, sensitivity, and fragility [1]. The actual treatment for patients with AI consists of covering teeth in the anterior sector with direct or indirect composite restorations and with stainless-steel crowns in the permanent first molars [2]. To follow a therapeutic gradient, minimally invasive dentistry should be the first choice to prevent pain, to enable harmonious facial skeletal growth, and to achieve an orthodontic treatment. This study aimed to present the application of the composite injection moulding technique for the anterior rehabilitation of a young patient with AI prior to orthodontic treatment.

## Case presentation

A 13-year-old girl was referred from the Department of Pedodontics to the Department of Prosthodontics with a diagnosis of AI. The patient has a history of early treatment with direct composite and orthodontic consultation at the same hospital. The chief complaints were anterior open bite, esthetic concern (patient unhappy with the shape and discoloration of her teeth), and tooth sensitivity. The family history revealed that her brother suffers from a severe form of AI. An extraoral examination showed a symmetrical face, incompetent lips, and a proportionally long lower anterior facial height.

**Figure 1:**
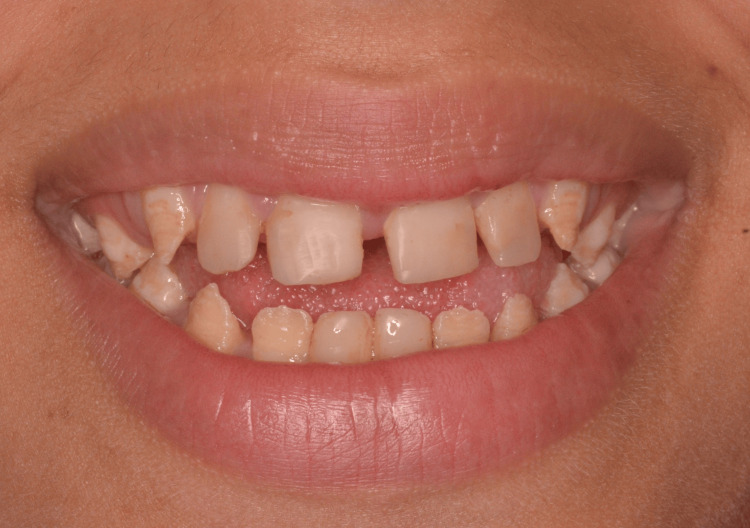
Extraoral preoperative smile.

A full smile was difficult to obtain. Her smile displayed an average smile line, a concave curvature of the maxillary incisors, and existing direct composite restorations on the maxillary incisors. The maxillary permanent canines were partially erupted (Figure 1).

**Figure 2:**
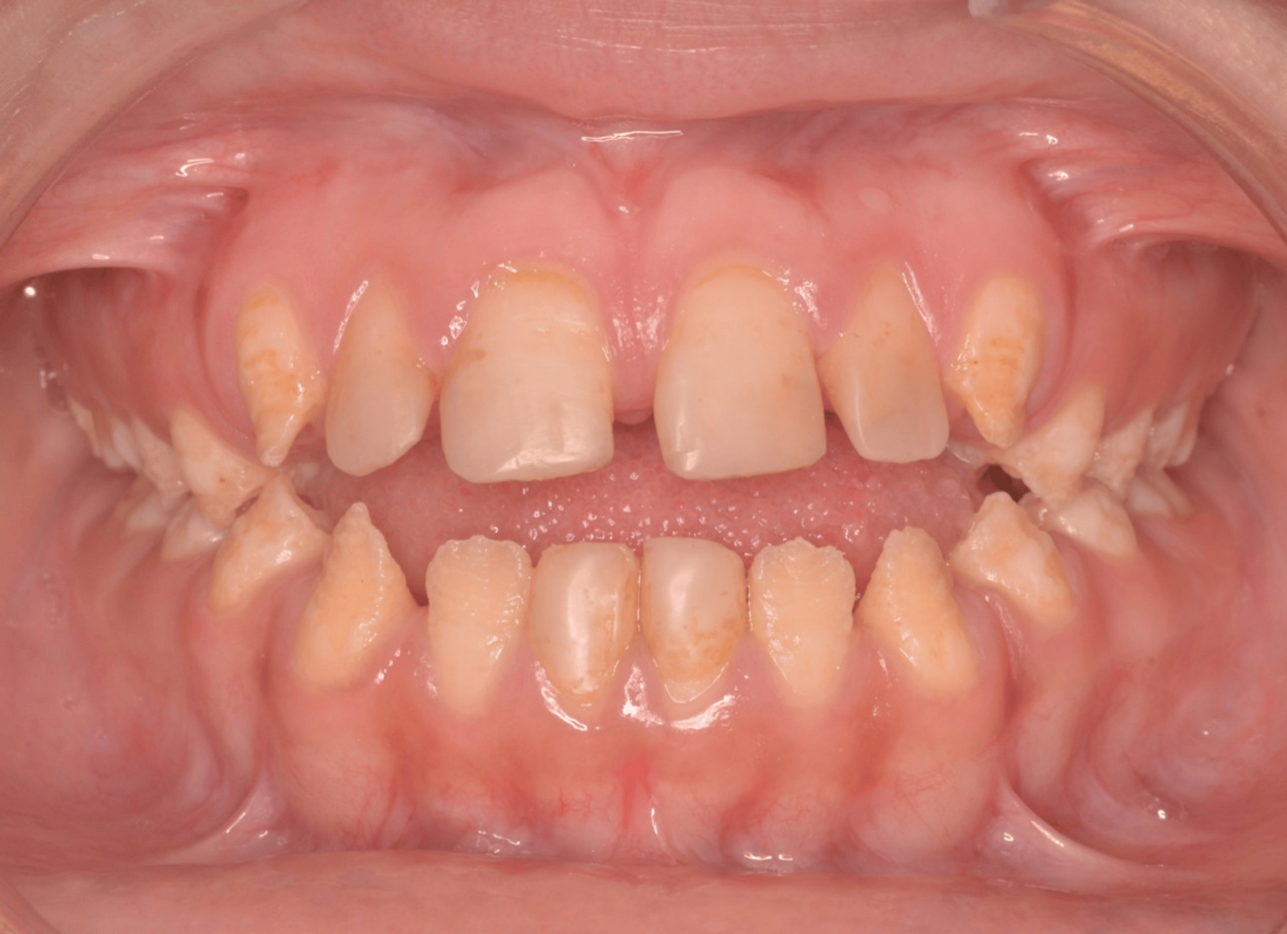
Intraoral anterior preoperative view.

Intraoral examination showed a class III and open bite skeletal discrepancy. The teeth presented staining and roughness and showed dentine exposed areas (Figure 2). The patient presented the characteristics of hypoplastic AI, such as reduced thickness of enamel, pitting and grooves, and tooth discoloration.

To improve the patient's psychological well-being, the treatment objectives were to correct her esthetic appearance and to facilitate the implementation of orthodontic treatment. Special consideration was required for the condition of brittle dentin, such as selecting the treatment modality and materials for bonding, because of insufficient adhesion to the superficial enamel. A restorative treatment of the anterior teeth with flowable charged composite via the injection molding technique was proposed.

**Figure 3:**
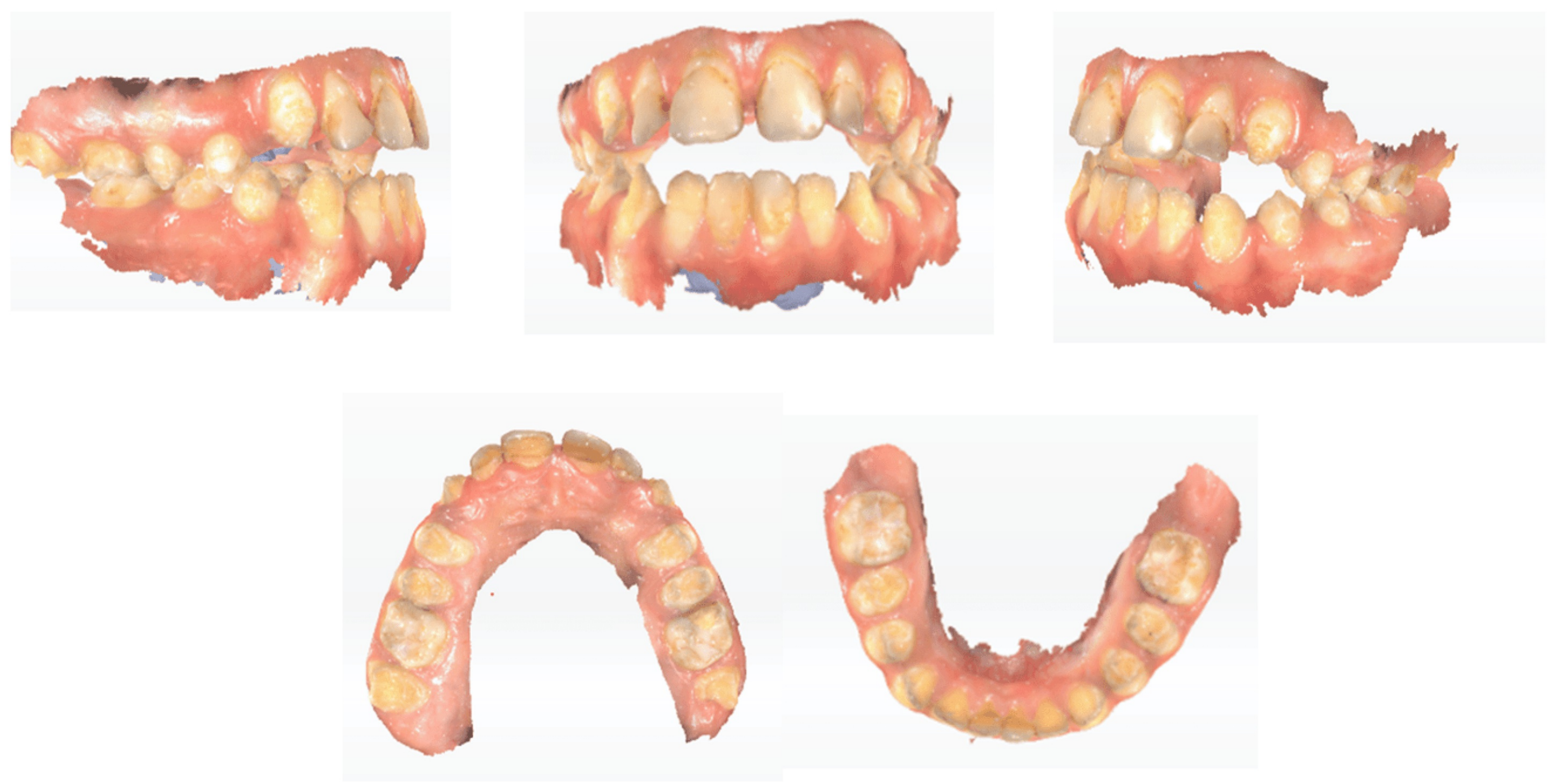
Frontal, lateral, and occlusal views of preoperative intraoral scan (STL files). STL: Standard Tessellation Language

The restoration of tooth integrity was made in stages. Treatment was initiated on the anterior teeth, and the upper four permanent incisors were restored with a flow composite using the composite injection technique, for the correction of the overall esthetic appearance of the teeth. Then, the posterior teeth were left for last. And, to achieve a correct occlusion, a clear aligner therapy (CAT) will be applied in the orthodontic department. The treatment plan sequence was as follows: maxillary and mandibular full arch digital impressions were made with a 3D intraoral scanner, Aoralscan 2 (Hangzhou, China: SHINING 3D Tech. Co., Ltd.) (Figure 3).

**Figure 4:**
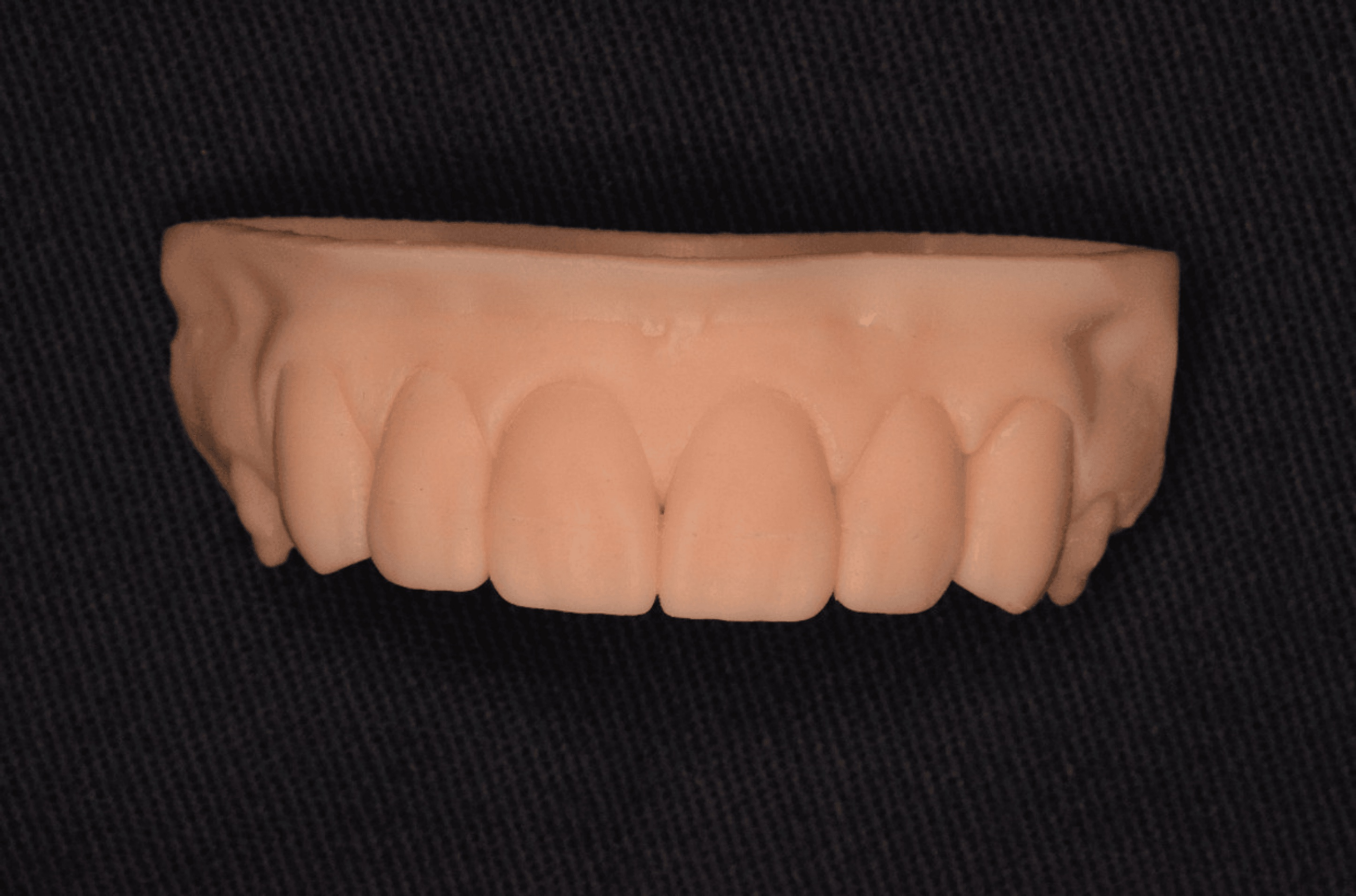
Three-dimensional printed model with DentaModel Asiga resin of the digital anterior wax-up.

A digital wax-up was made for the six anterior maxillary teeth with DentalCAD 3.1 Rijeka software (Darmstadt, Germany: exocad GmbH) followed by a 3D printed model with DLP printer (New South Wales, Australia: Asiga) (Figure 4).

**Figure 5:**
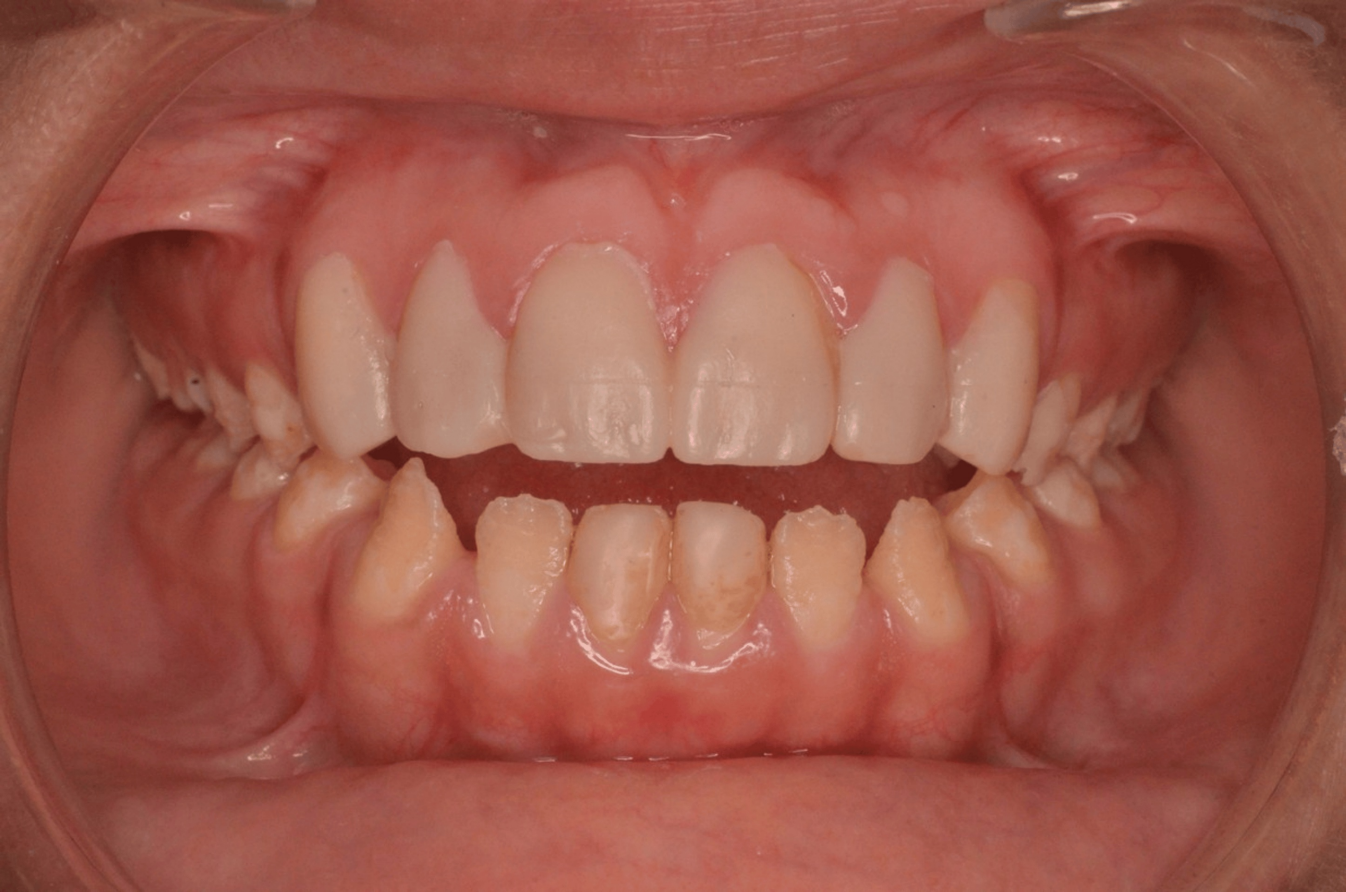
Intraoral mock-up simulating the final result.

**Figure 6:**
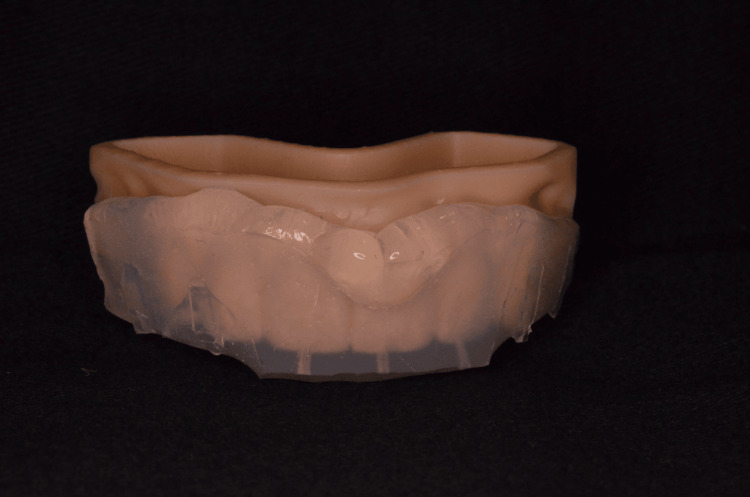
Transparent silicon index with openings for each tooth for composite injection.

To visualize esthetic results, a mockup with bis-acrylic resin (Protemp; Saint Paul, MN: 3M ESPE) was made, and the patient was satisfied with the esthetic aspect, although the open bite remained and will be solved by CAT (Figure 5). The composite injection molding technique was accomplished by using a transparent silicon index, with no tooth preparation except for the previous composite which was superficially removed (Figure 6).

The transparent silicon Exaclear (Tokyo, Japan: GC International AG) was dispensed in a non-perforated metal tray in the anterior sector with a minimum thickness (3-4 mm) to avoid distortions. On this transparent silicon index, we created a hole or a perforation for each tooth for the injection of the composite on the tips of the canine cusps and on the middle of the free margin of the incisors. With the pre-existing mock-up in place, we removed successively the mock-up from teeth number 13, 12, and 11, and we performed the injected composite technique one tooth after the other.

Isolation with a rubber dam was difficult to achieve, so we used polytetrafluoroethylene (PTFE) on the adjacent teeth with a mock-up. The tooth surfaces (mainly enamel) were etched with 35% phosphoric acid for 15-30 s, then rinsed and slightly dried. A bonding agent, Scotchbond Universal (Saint Paul, MN: 3M), was applied and light cured on the tooth.

**Figure 7:**
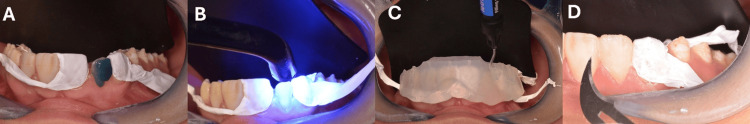
Operative procedures lateral incisor. (A) Etching of the labial surface of the lateral incisor with phosphoric acid. (B) Light curing after the application of adhesive. (C) Injection of the flowable composite resin through the occlusal opening of the index. (D) Removing excess of composite with a blade before polishing.

**Figure 8:**
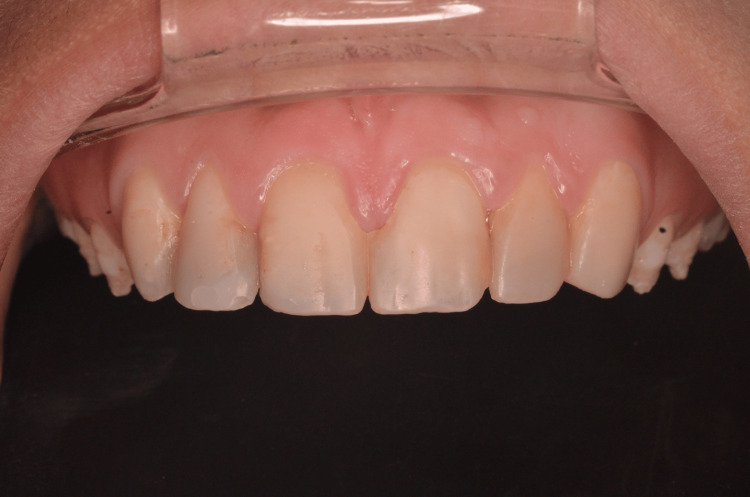
Postoperative intraoral view after polishing.

**Figure 9:**
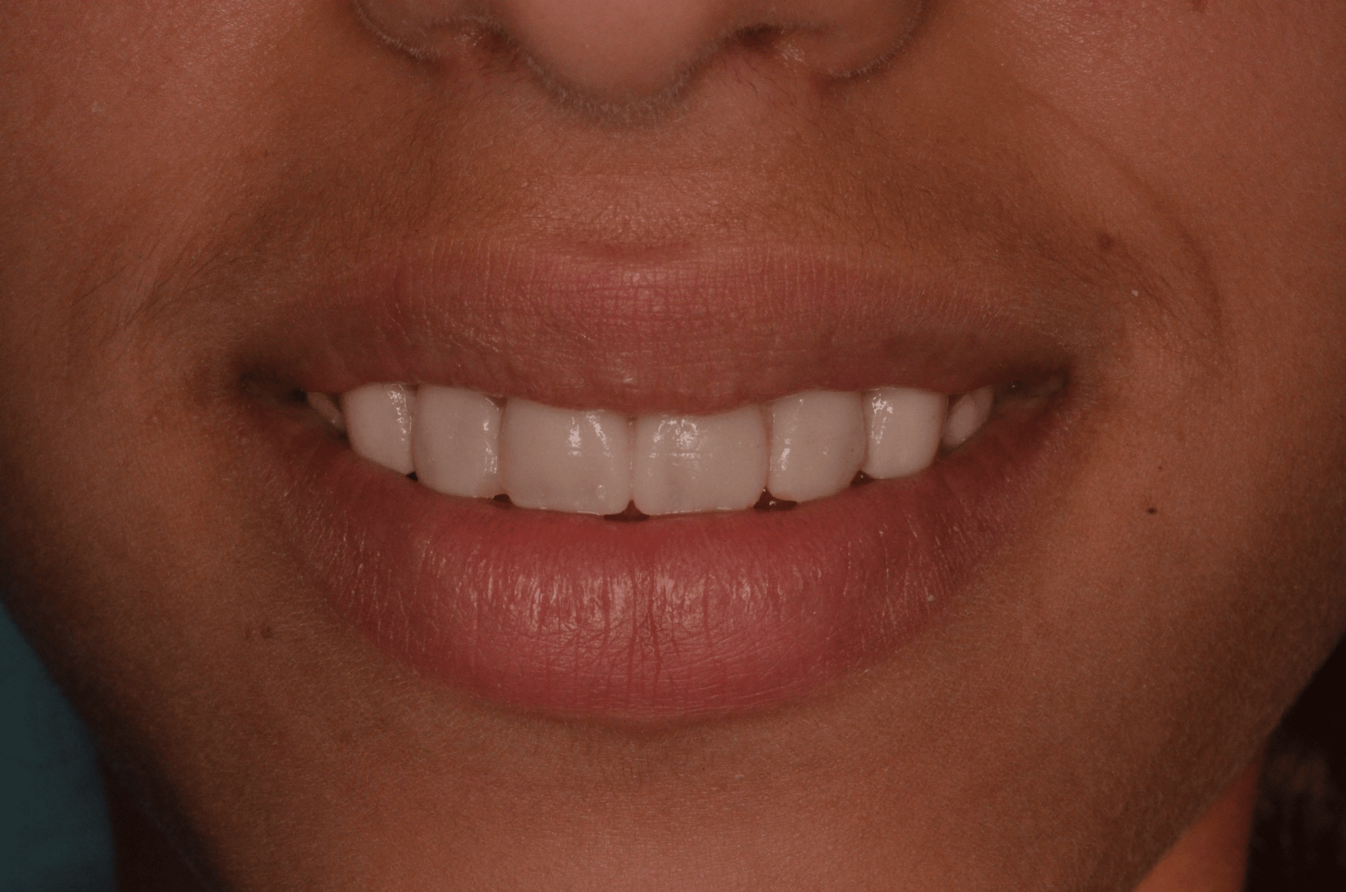
Patient smile after complete composite resin restoration of the anterior teeth similar to the 3D printed model.

We inject the G-ænial Universal Injectable composite (Tokyo, Japan: GC International AG) for the first tooth #13 until the excess of composite comes out from the hole while taking off the composite syringe tip. Each side was light cured for 20 s and the process was repeated for the other teeth successively and the other half of the anterior arch. Excess composite was removed with scalpel (Figures 7A-7D). All restorations were finished and polished with discs (SofLex; Saint Paul, MN: 3M ESPE) and rubber polisher (Figures 8, 9).

## Discussion

Children and adolescents with AI presenting anatomic loss may also present malocclusion, and it is therefore essential that a multidisciplinary management involves pediatric dentists, orthodontists, oral surgeons, oral and maxillofacial surgeons, and restorative dentists [1]. In our case, the anterior sector presented a dentin exposure with loss of incisor height and shape, and anterior open bite. The damaged areas were restored to their original dimensions and shape. The treatment with the composite injection molding technique is a conservative approach that helped meet the esthetic expectations of this patient, prior to orthodontic treatment and the necessity to ensure sufficient structural support for the future placement of fixed orthodontic appliances [3].

The orthodontist’s role usually begins in the late mixed dentition [4]. The application of clear aligner therapy (CAT) in patients with AI is favorable over fixed appliances. CAT includes a diverse selection of appliances that operate through different mechanisms, are manufactured using various techniques, and are compatible with multiple malocclusion treatment approaches. However, the existing evidence on the use of CAT in artificial intelligence is limited and disparate [5]. The composite restorative material is commonly used to repair defective structures due to its favorable esthetics and durability. It also provides suitable conservative interim therapy for AI protection of affected teeth [3].

The main advantage of the composite injection technique was the realization of virtual diagnostic waxing and the resulting 3D printing model. The digital previsualized esthetic proposal improved the predictability of the results. Visualization of the final prosthetic plan and type of AI must also be considered during orthodontic therapy [6]. For the posterior teeth, we intend to place stainless steel crowns as they are often placed on permanent first molars and restorative composite on the premolars [2].

Regarding prosthetic treatment, a significant number of anterior and posterior teeth require substantial coronal restoration [7]. The resin composite restorations otherwise used in ages 12-20 years often failed and needed to be replaced, increasing total costs [8]. However, the composite injection moulding technique is a viable alternative as biological and financial costs are lower, especially before an orthodontic treatment.

## Conclusions

In young patients with AI, all types of restorative therapy decreased symptoms of hypersensitivity and improved functional and esthetic issues. Early diagnosis and proper management at a young age provide successful rehabilitation.

The composite injection technique for anterior teeth restoration offers an optimal interim restorative solution until skeletal growth is complete. After orthodontic treatment, the patient will undergo complete rehabilitation with ceramic crowns.
